# Single-cell transcriptomic profiling of peripheral blood mononuclear cells reveals monocyte heterogeneity in patients with Moyamoya disease

**DOI:** 10.1186/s13023-026-04241-5

**Published:** 2026-02-05

**Authors:** Jinlin Xiao, Liwen Wei, Xingpeng Qiu, Jian Yan, Youping Li, Jinjing Wu, Haizhou Miu, Shuhua Zhang, Daya Luo, Erming Zeng

**Affiliations:** 1 Department of Neurosurgery, The First Affiliated Hospital, Jiangxi Medical College, Nanchang University, Nanchang, Jiangxi 330006 China; 2 Department of Neurosurgery, Pingxiang People’s Hospital, Pingxiang, Jiangxi 337000 China; 3 School of Basic Medical Sciences, Jiangxi Medical College, Nanchang University, Nanchang, Jiangxi 330006 China; 4 Jiangxi Cardiovascular Research Institute, Jiangxi Provincial People’s Hospital, The First Affiliated Hospital of Nanchang Medical College, Nanchang, 330006 China; 5 Nanchang University Affiliated Rehabilitation Hospital, Nanchang, 330006 China

**Keywords:** Moyamoya disease, Peripheral blood mononuclear cells, Monocytes, Single-cell sequencing

## Abstract

**Objective:**

Moyamoya disease (MMD) is a rare cerebrovascular disorder characterized by progressive stenosis or occlusion of the internal carotid artery, with an abnormal vascular network forming as compensation. The etiology of MMD remains largely unknown, though genetic and immune factors have been implicated. This study aimed to investigate the landscape of peripheral immune cells in MMD patients using single-cell RNA sequencing (scRNA-seq) to identify potential biomarkers and mechanisms involved in the disease.

**Methods:**

Peripheral blood mononuclear cells (PBMCs) were collected from six MMD patients and three controls. scRNA-seq was performed to analyze the transcriptomic profiles of various immune cell populations. Differential gene expression, functional enrichment, and cell interaction analyses were conducted to identify significant alterations in immune cell subpopulations. Additionally, trajectory analysis was used to explore the differentiation pathways of monocytes in MMD.

**Results:**

The study identified significant transcriptional alterations in peripheral immune cells, particularly in monocytes and natural killer (NK) cells. Notably, intermediate monocytes (Mono_CD14_CD16) were increased in MMD patients compared to controls. Functional enrichment analysis revealed upregulation of genes related to immune cell activation and signal transduction in MMD. Two previously uncharacterized genes, RETN and TGFBR2, were identified as potential biomarkers. Trajectory analysis suggested that classical monocytes may differentiate into intermediate monocytes in MMD. Cell interaction analysis highlighted the role of Mono_CD14_CD16 cells in mediating immune responses through interactions involving RETN and TGF-β signaling pathways.

**Conclusions:**

This study provides a comprehensive analysis of peripheral immune cell alterations in MMD, highlighting the involvement of monocyte subpopulations and specific signaling pathways in disease pathogenesis. The findings offer new insights into the immune dysregulation in MMD and suggest potential targets for diagnosis and treatment.

**Supplementary Information:**

The online version contains supplementary material available at 10.1186/s13023-026-04241-5.

## Introduction

Moyamoya disease (MMD) is a rare cerebrovascular disease characterized by the progressive stenosis or occlusion at the end of the bilateral internal carotid artery, which is associated with the formation of an abnormal vascular network [1]. Currently, the precise etiology of MMD is not fully understood. While prior research has identified the ring finger protein 213 (RNF213) gene as a potential susceptibility gene for MMD, other elements such as immune system dysregulation and inflammation have also been suggested as possible contributors to the onset and progression of the disease [2, 3].

Numerous studies have underscored the relationship between dysregulation of the peripheral immune system and MMD. The manifestation of MMD is frequently associated with autoimmune disorders, including type 1 diabetes, thrombocytopenia, and Graves’ disease [4–6]. Furthermore, patients diagnosed with MMD exhibit abnormal expression of inflammatory mediators and immune-related proteins in their peripheral blood. Research conducted by Gary K. Steinberg et al. identified 165 autoantibodies that are overexpressed in the serum of MMD patients through proteomic analysis [7]. Additionally, several investigations have reported elevated serum levels of interleukin-1 beta (IL-1β), tumor necrosis factor-alpha (TNF-α), and transforming growth factor-beta (TGF-β) in individuals with MMD [8]. Zhao et al. conducted a comparative analysis of peripheral immune cells in early-stage versus late-stage MMD patients, revealing that disease progression correlates with dysregulation in the proportions of various immune cell types. Notably, various immune cells exhibited abnormal activation of the NF-κB signaling pathway [9]. Prior research has also indicated that an imbalance in T cell populations, particularly a reduction in effector T cells alongside an increase in regulatory T cells (Treg), is closely linked to the pathogenesis of MMD [10]. Nevertheless, a comprehensive characterization of peripheral immune cells in the context of MMD remains insufficiently explored.

In this study, we conducted single-cell RNA sequencing to analyze the landscape of innate immune cells within peripheral blood mononuclear cells (PBMCs) from patients with MMD and normal controls. Our investigation focused on the transcriptomic characteristics of various immune cell populations, revealing significant alterations in peripheral monocytes among MMD patients. Additionally, resistin (RETN) and transforming growth factor beta receptor 2 (TGFBR2) were identified as candidate genes of interest in the PBMCs of this MMD cohort. These findings contribute to a deeper understanding of the mechanisms underlying MMD and offer novel perspectives for its diagnosis and therapeutic approaches.

## Materials and methods

### Sample collection, cell capture, and library construction

All six patients included in the study were recruited from the Department of Neurosurgery at the First Affiliated Hospital of Nanchang University, as detailed in Supplementary Table 1. The diagnosis of MMD was based on the 2021 Japanese guidelines. The study received approval from the Institutional Ethics Review Board, and informed consent was obtained from all of the participants. Control samples were selected from the public dataset GSE213516. Three samples that matched the patient group in both age and sex were chosen for downstream analysis [11]. Peripheral venous blood samples were collected in the morning from patients with MMD. Prior to the blood collection, all subjects had fasted for at least 12 h. PBMCs were isolated from the blood samples using density gradient centrifugation. The resulting single-cell suspension was subsequently processed using the 10X Genomics Chromium Chip, in accordance with the manufacturer’s instructions for the 10X Genomics Chromium Single-Cell 3’ kit, which included cDNA amplification and library construction. The prepared libraries were then sequenced on the Illumina NovaSeq 6000 platform using a paired-end approach with a read length of 150 base pairs. Single-cell cohort data from peripheral blood of patients with pulmonary arterial hypertension were obtained from GSE228643 [12], while data from patients with atherosclerosis were sourced from GSE253903 [13]. Furthermore, carotid artery tissue microarray data related to MMD were acquired from GSE157628 [14].

### Single cell data acquisition and tissue microarray data analysis

Initial read processing was performed using the Cell Ranger single-cell software suite (version 7.0.1, 10X Genomics, California, USA). The CellRanger mkfastq command was used to generated FASTQ files, and the CellRanger count command was used for primary data analysis, encompassing alignment, filtering, barcode counting, and UMI quantification to determine gene transcription counts per cell. Gene annotation was conducted with Ensembl build 93. After acquiring count expression matrix, Seurat package was used to conduct downstream analysis [15]. Cells were filtered to include only those with UMI counts from 1000 to 25,000, feature counts between 500 and 6000, and mitochondrial gene expression less than 20% of the total. The RPCA method was applied to correct for batch effects between samples.

### Differential gene expression analysis and functional enrichment

To identify genes exhibiting differential expression, we utilized the FindMarkers function. The criteria for the selection of differentially expressed genes (DEGs) included a minimum log2(fold change) of ≥ 1 and a FDR-adjusted P-value of ≤ 0.05. The scRNAtoolVis package was employed to visualize the results of the differential expression analysis (for further details, refer to the GitHub repository scRNAtoolVis). For the subcluster analysis of B cells, T/NK cells, and myeloid cells, we applied the RunHarmony function from the harmony package to perform batch correction [16]. Subsequently, the FindAllMarkers function conducted differential expression analysis on each cell subcluster, selecting genes with an absolute logFC of ≥ 0.25 and a P-value of ≤ 0.05 for further functional enrichment analysis. The analysis of DEGs in carotid artery tissue microarray data was carried out using the limma package [17].

Functional enrichment analysis was conducted using the Metascape online tool [18]. To assess functional differences among various cell subpopulations, we performed Gene Ontology-Biological Process (GO-BP) enrichment analysis. The compareCluster function from the clusterProfiler R package was utilized for this enrichment analysis, followed by the application of the simplify function to remove redundant pathway entries with a similarity greater than 0.5 [19]. Pathways with a Benjamini-Hochberg (BH) corrected P-value of ≤ 0.05 were considered significantly enriched.

### Transcription factor analysis

The pySCENIC package with default parameter settings was used to analyze transcription factors across various cell subpopulations [20]. Co-expression networks were determined using the GENIE3. Candidate transcription factors were identified via the RcisTarget al.gorithm, and the activity of transcription factors within each cell was quantified by AUCell. Ultimately, the output from pySCENIC was subjected to analysis with the SCopeLoomR package to discern specific transcriptional regulators for each cell subpopulation.

### Cell trajectory analysis and cell interaction analysis

We utilized the destiny, CytoTRACE2, Slingshot, and Monocle3 packages to evaluate cell differentiation trajectory [21–24]. Initially, the DiffusionMap and DPT functions were employed to determine the positions of monocytes along developmental coordinates. The Slingshot function from the Slingshot package facilitated the construction of the differentiation trajectory. The CytoTRACE2 package was utilized to evaluate the differentiation potential of each monocyte subset. To identify genes associated with cell differentiation, the graph_test function from the Monocle3 package was applied. Genes exhibiting a q_value ≤ 0.05 and morans_I ≥ 0.05 were considered significant, and the results of the analysis were visualized using the Cluster GVis package. Furthermore, the cell chat package was employed to elucidate the interactions between each monocyte subset and other immune cells [25]. The findings were graphically represented through the netVisual_bubble and plotGene Expression functions.

### Statistical analysis

Data visualization was conducted using R language (version 4.3). Data from each group are presented as mean ± standard deviation (mean ± SD). For data that are normally distributed, T-tests or ANOVA tests were used for intergroup comparisons, whereas for non-normally distributed data, Wilcoxon tests or Kruskal-Wallis tests were applied. Spearman’s rank correlation coefficient was employed for correlation analyses. A P-value of ≤ 0.05 was taken to indicate a statistically significant difference. Significance thresholds were established at *p* < 0.05 (*), *p* < 0.01 (**) and *p* < 0.001 (***) to indicate various levels of statistical significance.

## Results

### Single-cell transcriptional profiling of peripheral immune cells in normal and MMD groups

In order to evaluate the atypical alterations in the middle cerebral arteries of patients with MMD, we conducted an analysis utilizing a publicly accessible tissue microarray dataset that included two samples from MMD patients and four normal control samples. Initially, we performed differential expression analysis between the two groups and conducted enrichment analysis of the differentially expressed genes. The results revealed significant enrichment of immune cell responses and cell adhesion processes in MMD, suggesting a connection between the pathogenesis of the disease and immune-related processes. (Supplementary Fig. 1a-c). Subsequently, we examined the variations in immune cell infiltration within the middle cerebral arteries. The results demonstrated a notable increase in the presence of CD8 T cells, macrophages, and dendritic cells in the MMD samples (Supplementary Fig. 1d), highlighting potential specific targets for future investigative efforts.

**Fig. 1:**
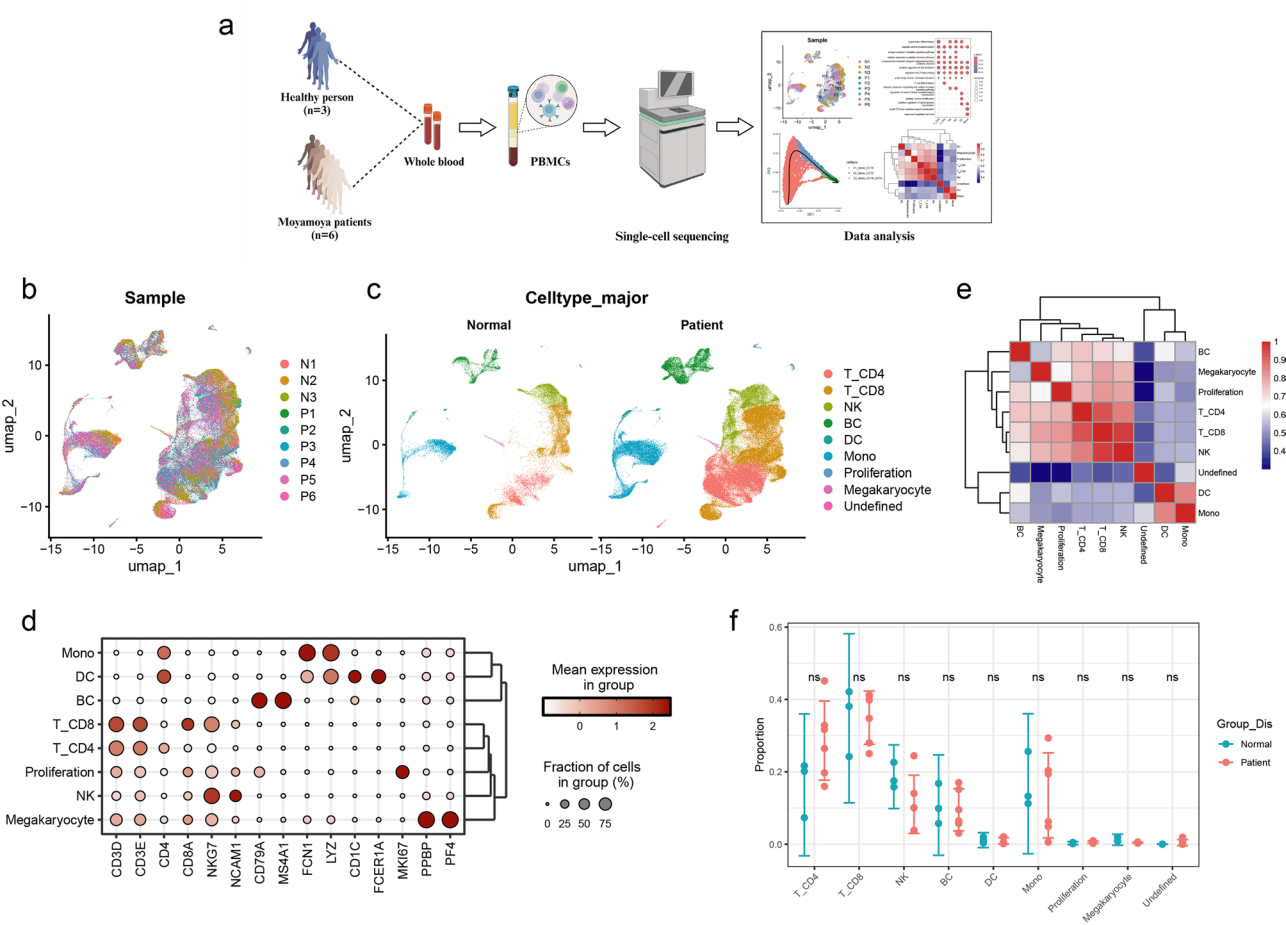
Comprehensive analysis of peripheral immune cells in normal controls and MMD patients. (**a**) Experimental design. (**b**) UMAP plot depicting cellular clustering from peripheral blood samples of three normal controls and six patients with MMD. (**c**) UMAP plots showing the distribution of 8 predominant cell types of normal controls and MMD patients. (**d**) Cell markers used to identify each cell subset. (**e**) Heatmap illustrating the correlation of transcriptional patterns among different cell types. (**f**) Comparative analysis of the differences in PBMC subset proportions between normal controls and MMD patients. Error bars represent the standard deviation. N, normal controls. P, patients

Next, we analyzed single-cell transcriptomic data derived from the PBMCs of three healthy individuals and six patients with MMD (Fig. 1a and b). Following a rigorous quality control process, we selected a total of 109,396 cells from MMD patients and 23,453 cells from normal controls for further examination (Supplementary Fig. 2a-d). After implementing batch effect correction, dimensionality reduction, and cell annotation, we identified six predominant cell types: CD4 + T cells (CD3E, CD4), CD8 + T cells (CD3E, CD8A), natural killer (NK) cells (NKG7, NCAM1), B cells (CD79A, MS4A1), dendritic cells (DCs) (CD1C, FCER1A), and monocytes (CD14, FCGR3A) (Fig. 1c). Specific cell markers were employed to ascertain and confirm the distribution of each cell subset (Fig. 1d). A comparative analysis of cell similarity revealed variations in transcriptional patterns among these cell types, thereby providing indirect validation of the accuracy of cell annotation (Fig. 1e). We subsequently assessed the relative proportions of each cell type, which revealed no statistically significant differences in subpopulation proportions between the normal controls and MMD patients (Fig. 1f). This finding suggests that alterations in cellular functional states may be more closely linked to the pathogenesis of MMD.

### Alterations in the functionality of peripheral immune cells in patients with MMD

**Fig. 2:**
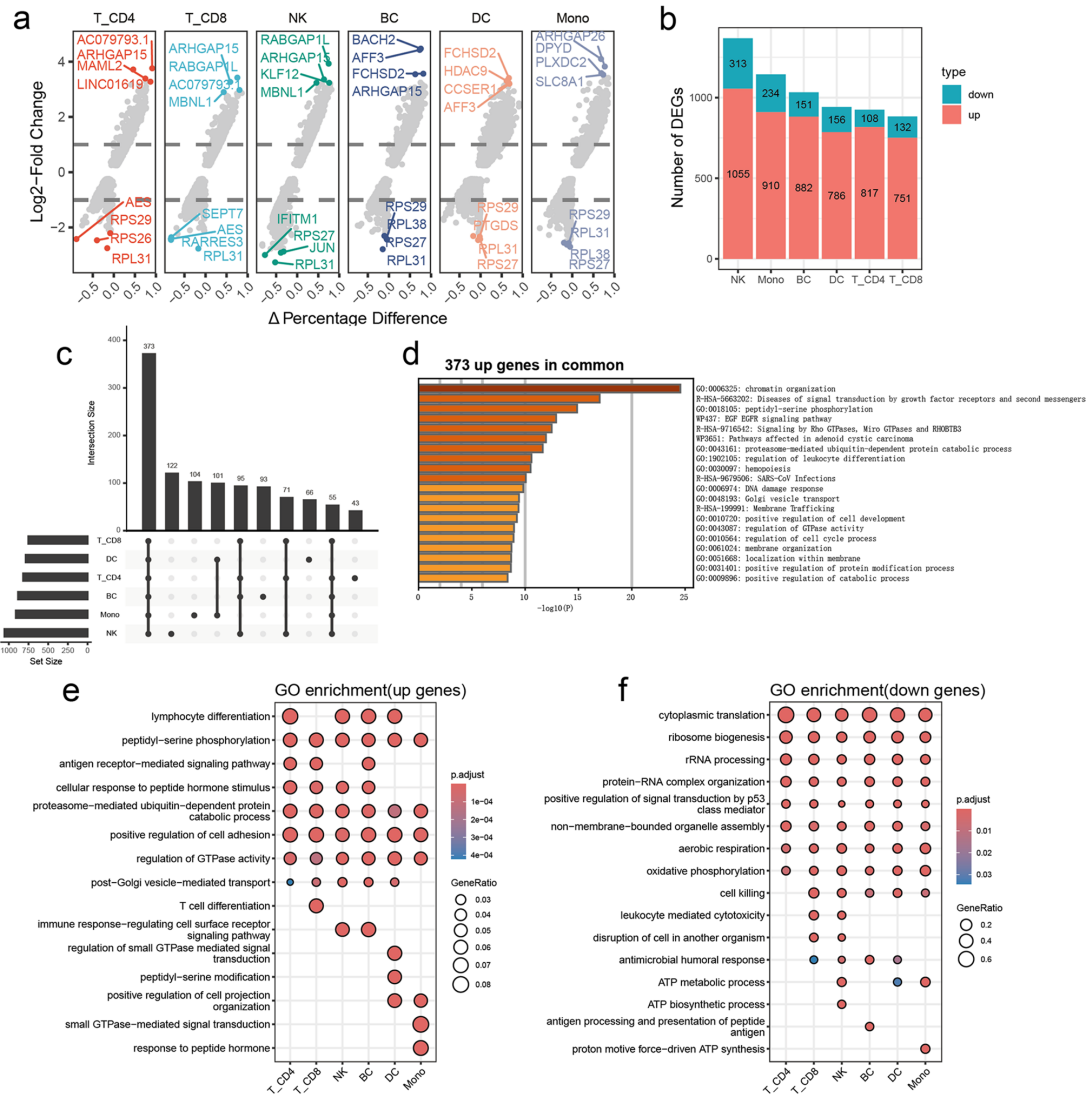
Transcriptomic profiling of different peripheral immune cells in MMD. (**a**) Comparative differential expression analysis of immune cells between the normal controls and MMD patients. (**b**) Statistical overview of DEGs across different immune cells. (**c**) Intersection analysis revealing upregulated genes for each cell type. (**d**) Functional enrichment analysis of 373 consistently upregulated genes. (**e**) Gene Ontology (GO) enrichment analysis of upregulated genes for each cell type. (**f**) Gene Ontology (GO) enrichment analysis of downregulated genes for each cell type

To deeply explore the transcriptomic alterations in immune cells of patients with MMD, we contrasted the expression profiles of MMD patients with normal controls across different peripheral immune cell lineages (Fig. 2a). Notably, NK cells exhibited the highest number of DEGs in MMD, followed by monocytes (Fig. 2b). This implies that NK cells and monocytes may be closely linked to the pathogenesis of MMD. Through gene functional enrichment analysis, we identified that 373 genes were consistently upregulated in the peripheral immune cells of MMD patients, with a predominant association to chromatin remodeling, the EGFR signaling pathway, leukocyte differentiation and protein synthesis (Fig. 2c and d). We further characterized the functional alterations in various peripheral immune cells of MMD patients. In CD4 + T cells, CD8 + T cells, and B cells, the DEGs that exhibited the most significant enrichment in MMD patients were related to lymphocyte differentiation and antigen receptor-mediated signaling (Fig. 2e). These findings indicate an abnormal activation of the adaptive immune system in MMD. In contrast, the upregulated genes in myeloid cells, such as DCs and monocytes, were enriched in signaling pathways related to signal transduction (Fig. 2e). Concurrently, the gene enrichment analysis of downregulated genes across various peripheral immune cells revealed that significant DEGs were involved in genomic maintenance, protein synthesis, and mitochondrial metabolism. These results imply that immune cells in MMD patients may experience genomic damage, impaired protein synthesis, and metabolic dysfunction (Fig. 2f). Collectively, these findings enhance our understanding of the transcriptomic alterations within the immune cell populations in MMD patients.

### Myeloid cell heterogeneity in normal controls and MMD patients

**Fig. 3:**
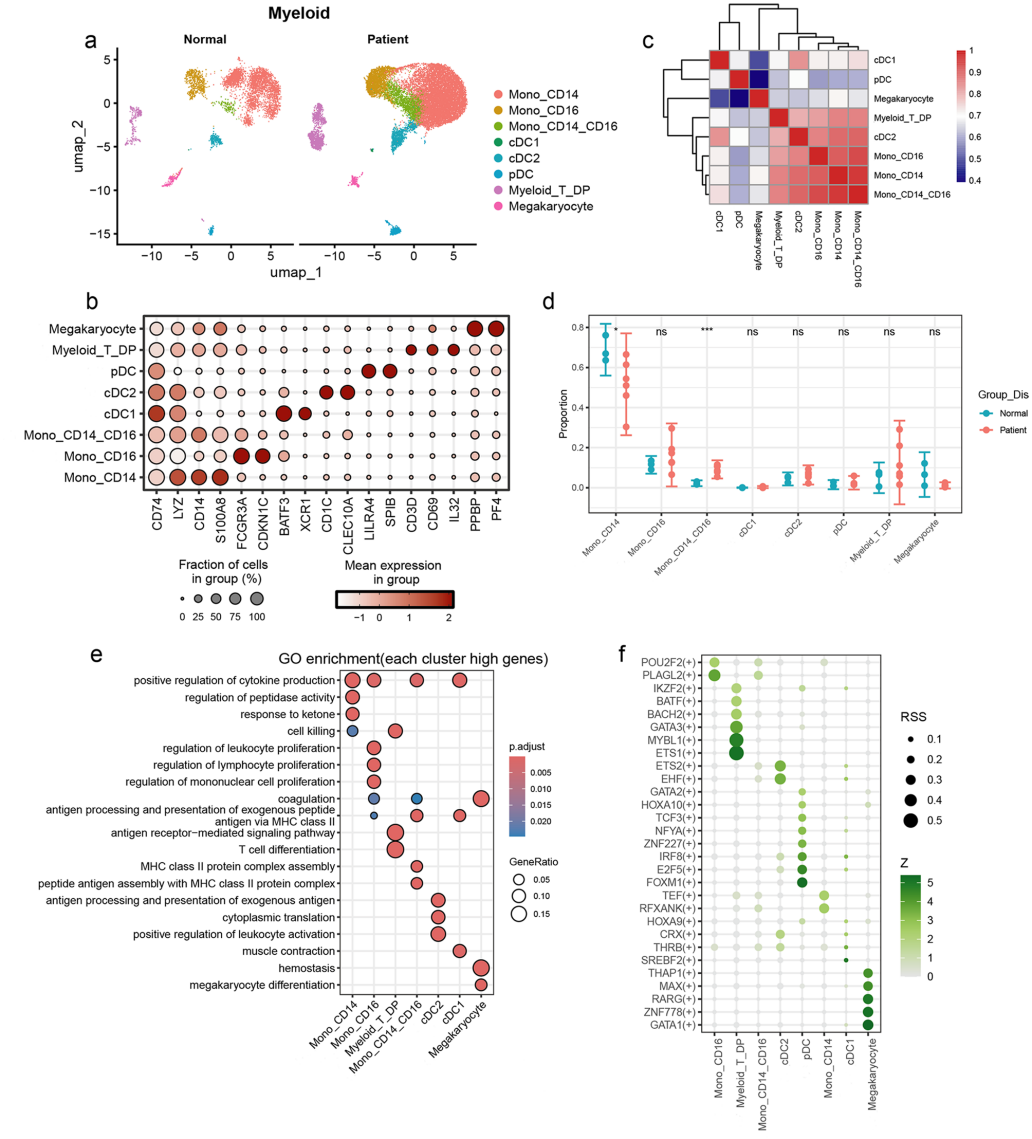
Single-cell analysis of peripheral myeloid cells in normal controls and MMD patients. (**a**) UMAP visualization depicting the clustering of peripheral blood myeloid cells in normal controls and MMD patients. (**b**) Heatmap illustrating the correlations of transcriptional patterns among diverse myeloid cell subpopulations. (**c**) Identification of highly expressed genes within each myeloid cell subpopulation. (**d**) Comparative analysis of the relative abundance of myeloid cell subpopulations between normal controls and MMD patients. Error bars represent the standard deviation. (**e**) Gene Ontology (GO) enrichment analysis highlighting highly expressed genes specific to each myeloid cell subpopulation. (**f**) Profiling of specifically activated transcription factors in various myeloid cell subpopulations

For a detailed exploration of the intrinsic functional changes in each myeloid cell subset, we selected 4,759 cells from the normal controls and 16,026 cells from the MMD patients for further analysis. We classified myeloid cells into eight subgroups, including classical monocytes (Mono_CD14, CD14 + S100A8+), nonclassical monocyte (Mono_CD16, FCGR3A+CDKN1C+), intermediate monocytes (Mono_CD14_CD16, CD14 + FCGR3A+), type 1 conventional DCs (cDC1, BATF3 + XCR1+), type 2 conventional DCs (cDC2, CD1C+CLEC10A+), plasmacytoid DCs (pDC, LILRA4 + SPIB+), T cell-like myeloid cells (Myeloid_T_DP, CD14 + CD3D+CD74+), and megakaryocyte (PPBP+PF4+) (Fig. 3a and b). The analysis of cell subset similarity analysis revealed that three monocyte subsets exhibited closely related transcriptional profiles (Fig. 3c). Notably, we identified an increased proportion of Mono_CD14_CD16 and a decreased proportion of Mono_CD14 in MMD patients compared to normal controls, while other myeloid cell populations did not demonstrate significant differences (Fig. 3d). Through analyzing scRNA-seq cohort of other cardiovascular diseases, we found that intermediate monocytes were also enriched in atherosclerosis and pulmonary arterial hypertension (Supplementary Fig. 3a-f). These results suggest that alterations in peripheral monocytes may significantly contribute to the pathogenesis of both MMD and other cardiovascular diseases.

Consequently, we conducted a gene functional enrichment analysis to clarify the specific alterations observed in various peripheral myeloid cell subpopulations in MMD patients. The findings indicated the following: (1) The Mono_CD14 subpopulation exhibited significant enrichment in pathways related to cytokine secretion, regulation of peptidase activity, and ketone response. (2) The Mono_CD16 subpopulation had a higher level of enrichment in cytokine secretion and lymphocyte proliferation. (3) The Mono_CD14_CD16 subpopulation showed a strong correlation with cytokine secretion and MHCII expression. Additionally, the cDC1 subpopulation was enriched in pathways associated with cytokine secretion and cellular contraction, while cDC2 was enriched in immune activation and antigen presentation pathways (Fig. 3E). We also investigated the variations in transcription factor activity across these cell subpopulations. The analysis revealed that the transcription factors PFXANK and TEF were notably activated in the Mono_CD14 subpopulation, whereas POU2F2 and PLAGL2 were preferentially induced in Mono_CD16. Additionally, EHF and ETS2 showed increased activity in cDC2, while ETS1, MYBL1, and GATA3 were induced in Myeloid_T_DP (Fig. 3f). In summary, our results highlight the heterogeneity and functional modifications of peripheral monocytes and DCs in MMD.

### T/NK cell heterogeneity in normal controls and MMD patients

As that transcriptional profiles of peripheral T cells and NK cells were similar, we next analyzed T/NK cell heterogeneity. We selected 15,880 T/NK cells from normal controls and 82,903 T/NK cells from the MMD patients. T/NK cells were classified into 14 distinct subpopulations, including naïve CD4 + T cells (T_CD4_naive, LEF1+), CD4 + central memory T cells (T_CD4_Tcm, ICAM2+), CD4 + exhausted T cells (T_CD4_Tex, PDCD1+), regulatory T cells (T_CD4_Treg, FOXP3+), naïve CD8 + T cells (T_CD8_naive, LEF1+), CD8 + central memory T cells (T_CD8_Tcm, ICAM2+), CD8 + effector memory T cells (T_CD8_Tem, GZMK+), CD8 + terminally differentiated T cells (T_CD8_Term, TCF7+), CD8 + tissue-resident memory T cells (T_CD8_Trm, ZEF683+), mucosal associated invariant T cells (T_CD8_MAIT, SLC4A10+), CD56^bright^ NK cells(NK_NCAM1, NKG7 + NCAM1+), CD56^dim^ NK cells (NK_FCGR3A, NKG7 + FCGR3A+), NK T cells (T_NK_DP, NKG7 + CD3D+), and myeloid-like T cells (T_Myeloid_DP, LYZ+FCN1+) (Supplementary Fig. 4a and b). Cell similarity analysis exposed the intrinsic correlations among these cell subpopulations (Supplementary Fig. 4c). Furthermore, we observed that T_CD4_Tcm, T_CD4_Tex, T_CD4_Treg, T_CD8_Term, and T_CD8_MAIT exhibited higher proportions in the MMD patients, whereas T_CD8_Tem and NK_FCGR3A displayed a lower abundance compared to normal controls (Supplementary Fig. 4d).

Through gene functional enrichment analysis, we found that each T/NK cell subpopulation exhibited immune-related signaling activation in MMD (Supplementary Fig. 4e). Lymphoid activation was enriched in T_CD4_Tcm and T cell differentiation was enriched in T_CD8_Tcm, T_CD4_naïve, T_CD8_MAIT and T_CD8_naïve. The analysis of transcription factors within each T/NK cell subpopulation revealed that the transcription factor ESR1 and SOX7 were specifically activated in T_CD4_naïve and T_CD4_Tex. SCAND1 was induced in NK_NCAM1, NK_FCGR3A, T_CD8_MAIT, T_CD8_Tem, and T_CD8_Trm. HOXA10 was activated in NK_NCAM1, NK_FCGR3A, T_CD8_Tem, and T_CD8_Trm. GATA3 was induced in T_CD8_Trm while RARB, USF1, and ZNF680 were activated in NK_NCAM1 (Supplementary Fig. 4f). In summary, our findings elucidate the heterogeneity present within peripheral T/NK cells in both normal controls and MMD patients.

### B cell heterogeneity in normal controls and MMD patients

To explore the function of peripheral B cell in MMD, we selected 2,740 B cells from normal controls and 9,326 B cells from MMD group. B cells were classified into seven distinct subsets, including pre-B cells (pre_B, AFF3 + PRKCE+), naïve cells (B_naive, TCL1A+IGHM+), mature B cells (mature_B, JCHAIN+), plasma (PDE4D+), memory B cells (B_memory, SOX5+), myeloid-like B cells (B_myeloid, LYZ+CD163+), and CD3D positive B cells (B_CD3D, CD3D+NKG7+) (Supplementary Fig. 5a and b). Cell similarity analysis disclosed that pre_B and B_myeloid cells exhibit unique transcriptional patterns (Supplementary Fig. 5c). Our investigation revealed a notable increase in pre-B cells among patients with MMD, accompanied by a significant enrichment in GTPase-mediated signal transduction pathways (Supplementary Fig. 5d-f). This finding underscores the potential role of pre-B cells in the pathogenesis of MMD. Furthermore, we observed an enrichment in protein synthesis within both naive B cells and mature B cells. The pathways associated with antigen processing and presentation, as well as B cell activation signaling, were found to be enriched in naive B cells, plasma cells, and memory B cells (Supplementary Fig. 5e). Subsequently, we analyzed the variations in transcription factor activity across different B cell subpopulations. The findings indicated that transcription factor ZGPAT was specifically activated in B_naive cells the andATF5 was augmented in mature_B cells. Plasma cells had an induced activation TCF3, POU6F1, HSF1, and ZNF821. SMAD1, VDR, FOSL2, and TCF7L2 were activated in B_myeloid cells activated (Supplementary Fig. 5f). Nevertheless, no distinct activation of transcription factors was observed in pre-B cells. In summary, our findings elucidate the diversity of peripheral B cells in both normal controls and MMD patients.

### The trajectory analysis of peripheral monocytes in MMD

**Fig. 4:**
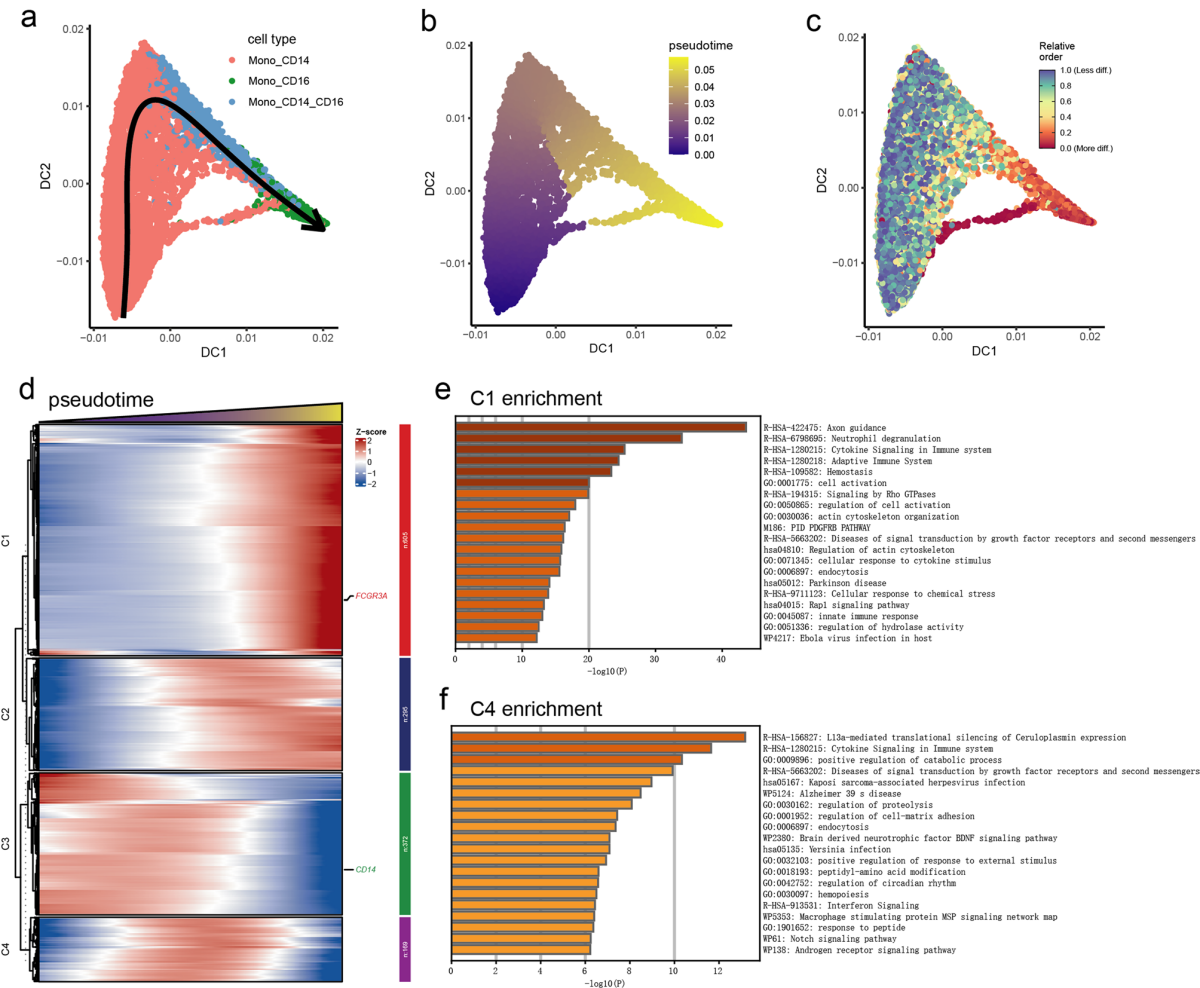
Trajectory analysis of three peripheral monocyte subsets in MMD. (**a**) Differentiation trajectories of peripheral monocyte subsets. (**b**) Pseudotime analysis of peripheral monocyte differentiation subsets. (**c**) Differentiation potentials of peripheral monocyte subsets. (**d**) Pseudotime heatmap showing the expression patterns of genes associated with the monocyte differentiation. (**e**) Functional enrichment analysis of genes within cluster C1. (**f**) Functional enrichment analysis of genes within cluster C4

Macrophages have been observed to infiltrate in the occlusive intracranial major arteries in MMD patients. This kind of macrophage may stem from the adhesion and differentiation of peripheral monocytes. Combined with our previous analysis of the proportions of peripheral immune cells, which indicated that monocytes in MMD exhibit significant transcriptomic alterations, we employed trajectory analysis to elucidate the developmental pathways of each monocyte subset in MMD. The findings suggest that Mono_CD14 in MMD may initially differentiate into Mono_CD14_CD16, which subsequently differentiates into Mono_CD16. The stemness scores of the three monocyte subsets corroborate this conclusion (Fig. 4a-c). To further identify genes associated with monocyte differentiation in MMD, we categorized these genes into four distinct clusters. The results revealed that the gene expression levels in clusters C1 and C2 progressively increased, while the genes in cluster C3 exhibited a decrease in expression during monocyte differentiation. In addition, the gene expression of cluster C4 initially increased and then subsequently decreased (Fig. 4d). The genes within Clusters C1 and C4, which may be linked to the differentiation of Mono_CD14_CD16, were found to be enriched in processes such as granulocyte degranulation, cytokine signaling, innate immune responses, and the Rap1 and Notch signaling pathways (Fig. 4e-f). However, monocyte trajectory analysis was restricted to MMD samples, as control monocytes formed distinct non-overlapping clusters with scant intermediate cells, providing no evidence of a continuous trajectory—a pattern uniquely observed in MMD (Supplementary Fig. 6a-d). In summary, these findings elucidate the differentiation trajectory of peripheral monocytes in MMD.

### Cell interaction analysis of peripheral monocytes in MMD

**Fig. 5:**
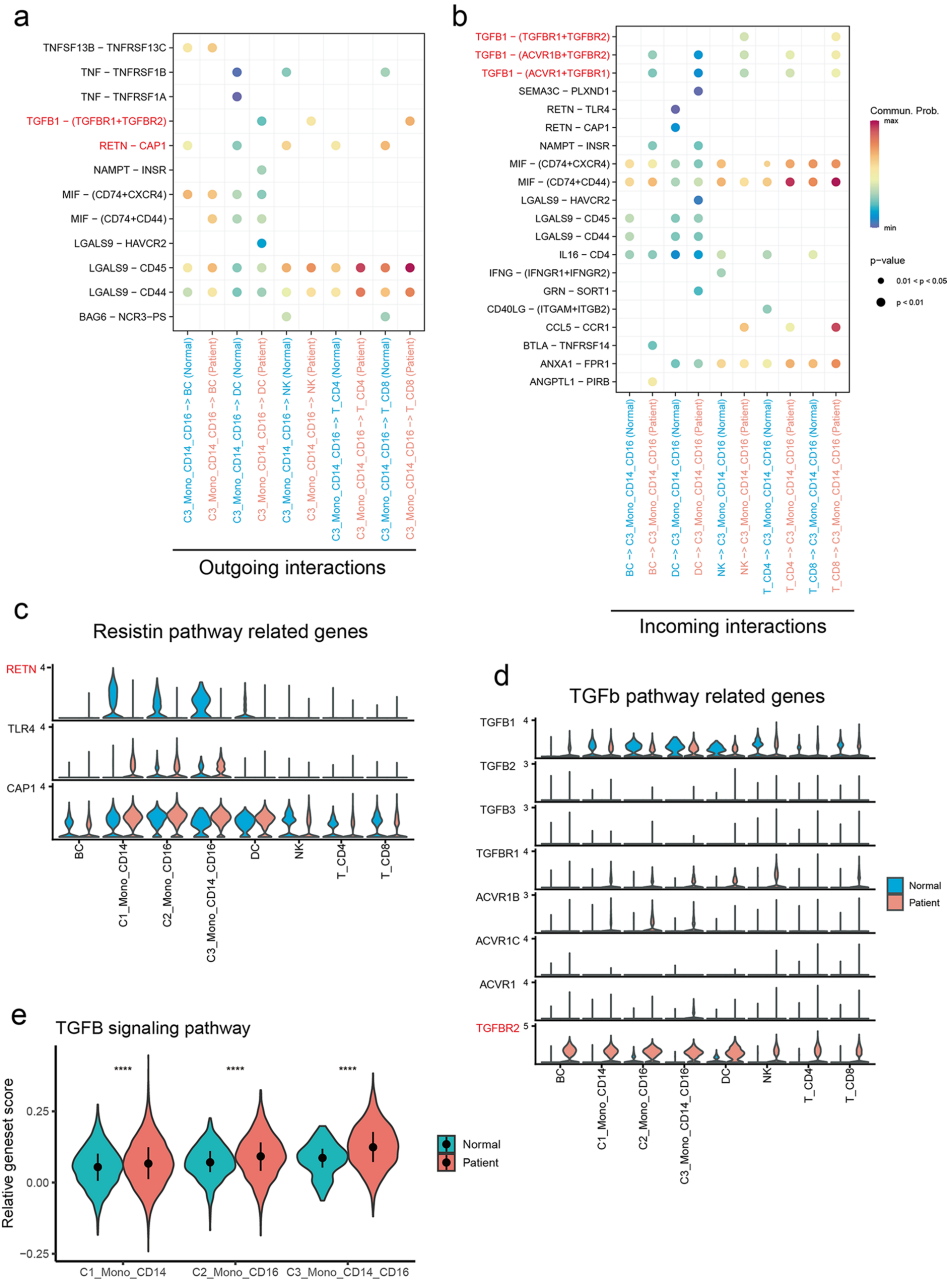
Cell interaction analysis of peripheral monocyte subsets and other immune cells. (**a**) The outgoing interactions between peripheral monocyte subsets and other immune cells. (**b**) The incoming interactions between peripheral monocyte subsets and other immune cells. (**c**) The expression of RETN pathway-related genes in the peripheral immune cells of normal controls and MMD patients. (**d**) The expression of TGF-β pathway-related genes in the peripheral immune cells of normal controls and MMD patients. (**e**) Violin plots showing the activity levels of the TGF-β signaling pathway across monocyte subsets in both groups (mean ± interquartile range)

To further clarify the role of peripheral Mono_CD14_CD16 cells in MMD, we conducted an analysis of cell-cell interactions. Firstly, we analyzed the role of Mono_CD14_CD16 cells acted as signal senders. The results showed that the TGFB1-(TGFBR1 + TGFBR2) and RETN-CAP1 pathways were implicated in the interactions between Mono_CD14_CD16 cells and other immune cells (Fig. 5a). Moreover, when Mono_CD14_CD16 cells were assessed as signal receivers, the TGFB1-(TGFBR1 + TGFBR2), TGFB1-(ACVR1B+TGFBR2), and TGFB1-(ACVR1B+TGFBR1) pathways were consistently enriched in the MMD (Fig. 5b). Subsequently, we investigated the expression levels of genes associated with RETN or the TGF-β pathway across different cell subsets between normal controls and MMD patients. Notably, we observed a reduction in the expression of RETN in peripheral monocytes, while TGFBR2 expression was elevated among various peripheral immune cells in MMD (Fig. 5c and d). These results imply that RETN and TGFBR2 may have critical roles in the pathogenesis of MMD, warranting further investigation.

### The expression pattern of MMD-related cytokines

**Fig. 6:**
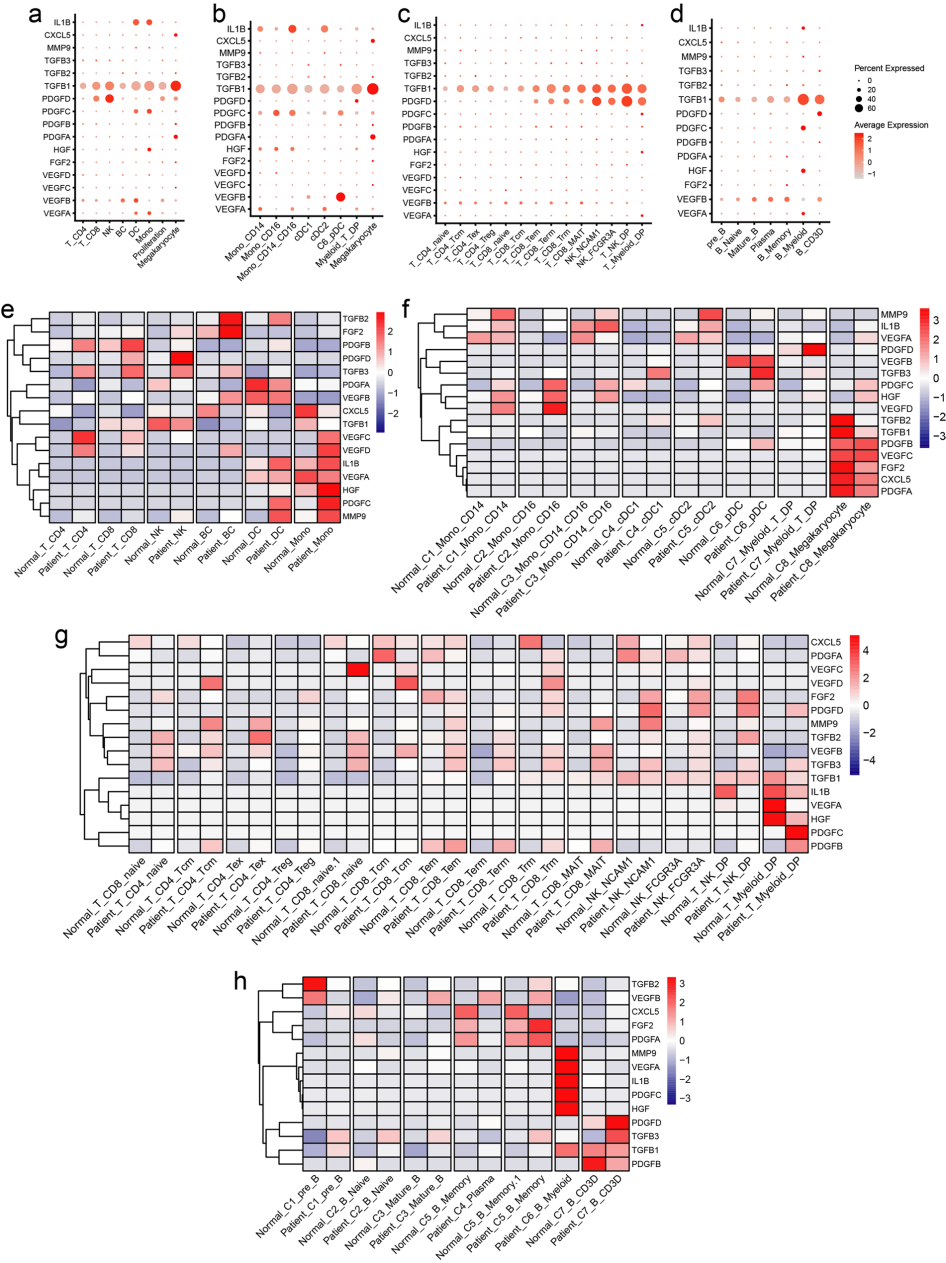
Expression profiles of MMD-associated cytokines in peripheral immune cells. (**a**-**d**) Bubble charts illustrating the expression levels of MMD-associated cytokines across various immune cells in peripheral blood. (**e**-**h**) Heatmaps depicting the comparative expression levels of MMD-associated cytokines in various immune cells in peripheral blood between normal controls and MMD patients

Recent studies have suggested that the dysregulated accumulation of various cytokines in peripheral blood may be associated with the pathogenesis of MMD. In this context, we employed single-cell transcriptomic analysis to elucidate the primary cellular sources of MMD-related cytokines. Our findings revealed that TGFB1 was predominantly expressed in megakaryocytes, monocytes, and NK cells, while IL1B was primarily produced by intermediate monocytes (Fig. 6a-d). Additionally, we conducted a comparative analysis of the differential expression of these signaling molecules in peripheral immune cells between normal controls and MMD patients. The heatmap indicated that MMP9 and PDGFC exhibited increased expression levels in peripheral monocytes and dendritic cells, whereas PDGFC and HGF showed heightened expression in both Mon_CD14 and Mon_CD16 subsets among MMD patients (Fig. 6e-h). In summary, our results elucidate the expression patterns of cytokines associated with MMD.

## Discussion

Currently, the precise pathogenesis of MMD remains elusive. Prior genomic investigations have identified several susceptibility genes linked to MMD, among which the most well-known is RNF213 [26]. While the RNF213 p.R4810K variant confers extremely high disease risk and has a carrier frequency of 0.5–2% in these populations, only approximately 0.5% of heterozygous carriers develop MMD. This indicates that additional genetic or environmental hits are necessary for disease manifestation [27, 28]. Numerous studies have underscored the association between MMD and immune dysregulation caused by autoimmune diseases and infection [29]. Studies have indicated that patients with MMD and Graves’ disease experience a significantly accelerated disease progression, implying that Graves’ disease may serve as an independent factor influencing the advancement of MMD in adult patients [30, 31]. Furthermore, patients who have suffered from bacterial meningitis due to various infectious agents are also at an elevated risk of developing MMD-like vascular lesions [32]. In the present study, we performed a comparative analysis of peripheral immune cells from MMD patients and normal controls, which revealed significant transcriptional alterations in the peripheral immune cells of the MMD cohort. Notably, 373 genes were consistently upregulated across various peripheral immune cells in the MMD group, implying that these patients exhibit marked peripheral immune dysregulation. Our findings, together with previous single-cell studies describing broad immune dysregulation and T-cell abnormalities in MMD [10, 45], collectively depict a more complex and comprehensive immune landscape involving both innate and adaptive immunity. Specifically, we expand the understanding of innate immune mechanisms by revealing the expansion and potential differentiation trajectory of intermediate monocytes, and identifying RETN and TGFBR2 as candidate genes. Furthermore, our cell-cell communication analysis highlights the specific role of RETN and TGF-β signaling pathways in mediating interactions, particularly through intermediate monocytes, thereby providing complementary insights to previously reported immune pathways and underscoring the cooperative involvement of diverse immune cell subsets in MMD pathogenesis.

Stenosis or occlusion caused by intimal thickening in the major intracranial arteries is recognized as the primary pathological feature in MMD. While intimal thickening in MMD is generally not characterized by inflammatory cell infiltration, certain studies have reported the presence of macrophages and T cell infiltration within the thickened intima of intracranial arteries in patients with MMD [33]. During the progression of MMD, these macrophages may originate from the migration and differentiation of circulating monocytes, thereby initiating and exacerbating further immune responses [34, 35]. Circulating monocytes exhibit heterogeneity and are divided in three major classes: classical (CD14 + CD16-), intermediate (CD14 + CD16+), and nonclassical (CD14-CD16+). Classical monocytes are involved in phagocytosis, innate immune sensing, immune responses, and migration. Intermediate monocytes are characterized by the highest expression levels of antigen presentation-related molecules and have been shown to secrete pro-inflammatory cytokines such as TNF-α, IL-1β and IL-6 upon Toll-like receptor (TLR) stimulation, which are crucial for rapid pathogen defense [36, 37]. Recent research has identified an increased prevalence of classical monocytes with elevated CD163 expression in the peripheral blood of MMD patients [38]. Another study reported heightened levels of non-classical monocytes, which exhibit enhanced adhesion and chemotaxis capabilities in the early-stage of MMD [9]. In our investigation, we noted an increased proportion of Mono_CD14_CD16 and a decreased proportion of Mono_CD14 in MMD patients compared to normal controls. Previous studies have indicated that intermediate monocytes can significantly rise during both acute and chronic inflammatory diseases. A cohort study revealed that the percentage of CD14 + CD16+ monocytes in the peripheral blood of patients with coronary artery disease was elevated and significantly correlated with the risk of developing the disease [39]. Furthermore, an increase in CD14 + CD16+ monocytes was also observed in patient with acute Kawasaki disease [40]. Consequently, we hypothesize that the elevation of intermediate monocytes may serve as an independent risk factor for various vascular diseases. The differentiation trajectory analysis of peripheral monocytes in our study further suggests that classical monocytes possess a greater differentiation potential and may initially transition into intermediate monocytes during the progression of MMD. Collectively, these findings indicate that intermediate monocytes accumulate in MMD patients, suggesting a state of systemic immune dysregulation.

Our study has elucidated abnormal ligand-receptor interactions involving RETN and TGF-β signaling in Mono_CD14_CD16 cells within MMD through an analysis of cell-cell interaction. Specifically, we observed decreased expression of RETN alongside increased expression of its receptor CAP1 in peripheral monocytes. Although RETN can exhibit both pro- and anti-inflammatory effects [41, 42], the functional consequences of its downregulation in MMD remain unclear. Based on our findings, we propose a “loss of brake” model wherein reduced RETN ligand expression, coupled with elevated CAP1 receptor levels, may disrupt a putative regulatory circuit in monocytes. This imbalance could potentially heighten cellular responsiveness to inflammatory stimuli, contributing to immune dysregulation in MMD. Further studies measuring plasma resistin levels and delineating the precise role of the RETN-CAP1 axis are warranted to validate this hypothesis. Subsequently, the TGFB1-(TGFBR1 + TGFBR2) signaling pathway appears to mediate the interactions between intermediate monocytes and other immune cells. Notable, we also noted a consistent upregulation of TGFB1 and TGFBR2 in the peripheral immune cells of MMD cohort. Previous studies have demonstrated that patients with MMD exhibit higher expression levels of TGFB1 compared to healthy controls, which is consistent with our findings [43]. TGFB1 is considered as a principal inducer of endothelial-to-mesenchymal transition (EndMT), a complex biological process wherein endothelial cells lose their specific phenotype and progressively transform into cells with a mesenchymal phenotype. Although the precise role of EndMT in the development of MMD is still unclear, it has been extensively studied in cardiovascular disease, where it contributes to the development of atherosclerosis, pulmonary hypertension and cardiac fibrosis [44]. These findings suggest that targeting molecules involved in the TGF-β signaling pathway may represent a promising therapeutic strategy for MMD.

Peripheral blood B and T lymphocytes are derived from hematopoietic stem cells located in bone marrow and undergo processes of activation, proliferation, and differentiation upon encountering antigen. Researches indicate that imbalance between pro-inflammatory and anti-inflammatory T cells may play a role in the pathogenesis of various cerebrovascular diseases [45]. In our study, we observed that the percentages of CD4 + Tcm, CD4 + Tex, Treg, and CD8 + Term were elevated, whereas CD8 + Tem were decreased in patients with MMD compared to normal controls. These observations are consistent with previous studies that have reported significant T cell abnormalities in PBMCs in MMD [10]. Furthermore, B cells are implicated in the pathogenesis and progression of various chronic inflammatory conditions through their role in presenting autoantigens and secreting pro-inflammatory cytokines. Our findings revealed an increased percentage of pre-B cells in MMD patients, which exhibited heightened expression of genes related to GTP signal transduction. Therefore, we propose that hematopoietic stem cells in the bone marrow may exhibit intrinsic abnormalities in differentiation and signaling pathways in the context of MMD. Collectively, our study contributes valuable insights to the existing body of research concerning peripheral immune cell dynamics in MMD.

The study presents several limitations. Firstly, the sample size is comparatively small, and the normal control data were sourced from a public database. Secondly, we did not perform cellular and clinical experiments to corroborate our analytical results, nor did we offer a thorough elucidation of the mechanisms in the progression of MMD. Our future research will aim to incorporate additional public single-cell transcriptomic data for a more comprehensive analysis and concentrate on exploring the roles of RETN and the TGF-β signaling pathway in MMD.

## Conclusion

Our research presents a thorough examination of the peripheral immune cell profile in patients with MMD, revealing significant alterations in both the proportion sand functionality of various immune cell subpopulations, particularly monocytes. These findings contribute valuable insights for the development of future diagnostic and therapeutic strategies for MMD.

## Electronic supplementary material

Below is the link to the electronic supplementary material.

Supplementary Material 1: Fig. 1: Abnormal Changes in the Middle Cerebral Arteries of Moyamoya Disease Patients. (a) A comparative differential expression analysis between the middle cerebral arteries of moyamoya disease patients and normal controls. (b) Functional enrichment analysis of the 310 upregulated genes in the middle cerebral arteries of moyamoya disease patients. (c) Functional enrichment analysis of the 377 downregulated genes in the middle cerebral arteries of moyamoya disease patients. (d) Differential expression of various immune cell markers between the middle cerebral arteries of moyamoya disease patients and controls. Con, controls. Moy, moyamoya disease.

Supplementary Material 2: Fig. 2: Peripheral Blood Single-Cell Data Preprocessing and Overview of Information. (a) Violin plots display the expression levels of UMI, Feature, and mitochondrial gene expression after quality control for each sample. (b) Feature plots illustrate the annotation markers for various cell types. (c) Absolute and relative proportions of various cell types across samples. (d) Violin plots of the platelet module score across cell types. (e-g) UMAP plots colored by sample origin. N, normal controls. P, patients.

Supplementary Material 3: Fig. 3: Analysis of Monocytes in Peripheral Blood in Pulmonary Artery Hypertension and Atherosclerosis. (a) and (d) Single-cell transcriptomic data from peripheral blood in patients with pulmonary artery hypertension and atherosclerosis. (b) and (e) Identification of heterogeneity in monocytes from peripheral blood in pulmonary artery hypertension and atherosclerosis. (c) and (f) Relative proportions of various monocyte subtypes in control and disease groups, including pulmonary artery hypertension or atherosclerosis. PHPF, pulmonary arterial hypertension. AS, atherosclerosis. cMo, classical monocytes. iMo, intermediate monocytes. ncMo, nonclassical monocytes.

Supplementary Material 4: Fig. 4: Single-Cell Analysis of Peripheral Blood T/NK Cells in Normal Controls and Moyamoya Disease Patients. (a) UMAP visualization of T/NK cell clustering in peripheral blood from normal and moyamoya disease groups. (b) Highly expressed genes across various T/NK cell subsets. (c) Heatmap of transcriptional pattern correlations among T/NK cell subsets. (d) Relative quantity differences of T/NK cell subsets between normal and moyamoya disease groups. Error bars represent the standard deviation. (e) GO enrichment analysis for highly expressed genes specific to each T/NK cell subset. (f) Specific activated transcription factors characteristic of various T/NK cell subsets.

Supplementary Material 5: Fig. 5: Single-Cell Analysis of Peripheral Blood B Cells in Normal and Moyamoya Disease Groups. (a) UMAP visualization of peripheral blood B cell clustering in normal and moyamoya disease groups. (b) Highly expressed genes in various B cell subsets. (c) Heatmap of transcriptional pattern correlations across B cell subsets. (d) Relative quantity differences of various B cell subsets between normal and moyamoya disease groups. Error bars represent the standard deviation. (e) GO enrichment analysis of highly expressed genes specific to each B cell subset. (f) Specific activated transcription factors in various B cell subsets.

Supplementary Material 6: Fig. 6: Trajectory analysis of monocyte differentiation in MMD and control groups. (a–d) UMAP projections showing the distribution and transitional continuum of monocyte subsets (CD14⁺, intermediate, and CD16⁺) in MMD patients (a, c) and healthy controls (b, d). Note the clear bridging population of intermediate monocytes in MMD, which is absent in controls. N, normal controls. P, patients.

Supplementary Material 7: Table 1: Characteristics of the study population of three normal controls and six patients with MMD.

## Data Availability

The data that support the findings of this study are available from the corresponding author upon reasonable request.

## Abbreviations

MMD: Moyamoya disease
scRNA-seq: Single-cell RNA sequencing
RNF213: Ring finger protein 213
IL-1β: Interleukin-1 beta
TNF-α: Tumor necrosis factor-alpha
TGF-β: Transforming growth factor-beta
Treg: Regulatory T cells
NK cells: Natural killer cells
DCs: Dendritic cells
PBMCs: Peripheral blood mononuclear cells
DEGs: Differentially expressed genes
RETN: Resistin
TGFBR2: Transforming growth factor beta receptor 2
GO-BP: Gene Ontology-Biological Process
BH: Benjamini-Hochberg
EndMT: Endothelial-to-mesenchymal transition
