# Supplementary figures and images for: Single-cell transcriptomic profiling of peripheral blood mononuclear cells reveals monocyte heterogeneity in patients with Moyamoya disease
Source: Orphanet J Rare Dis. 2026 Feb 5;21:93. doi: 10.1186/s13023-026-04241-5 (PMC12973665; doi:10.1186/s13023-026-04241-5)

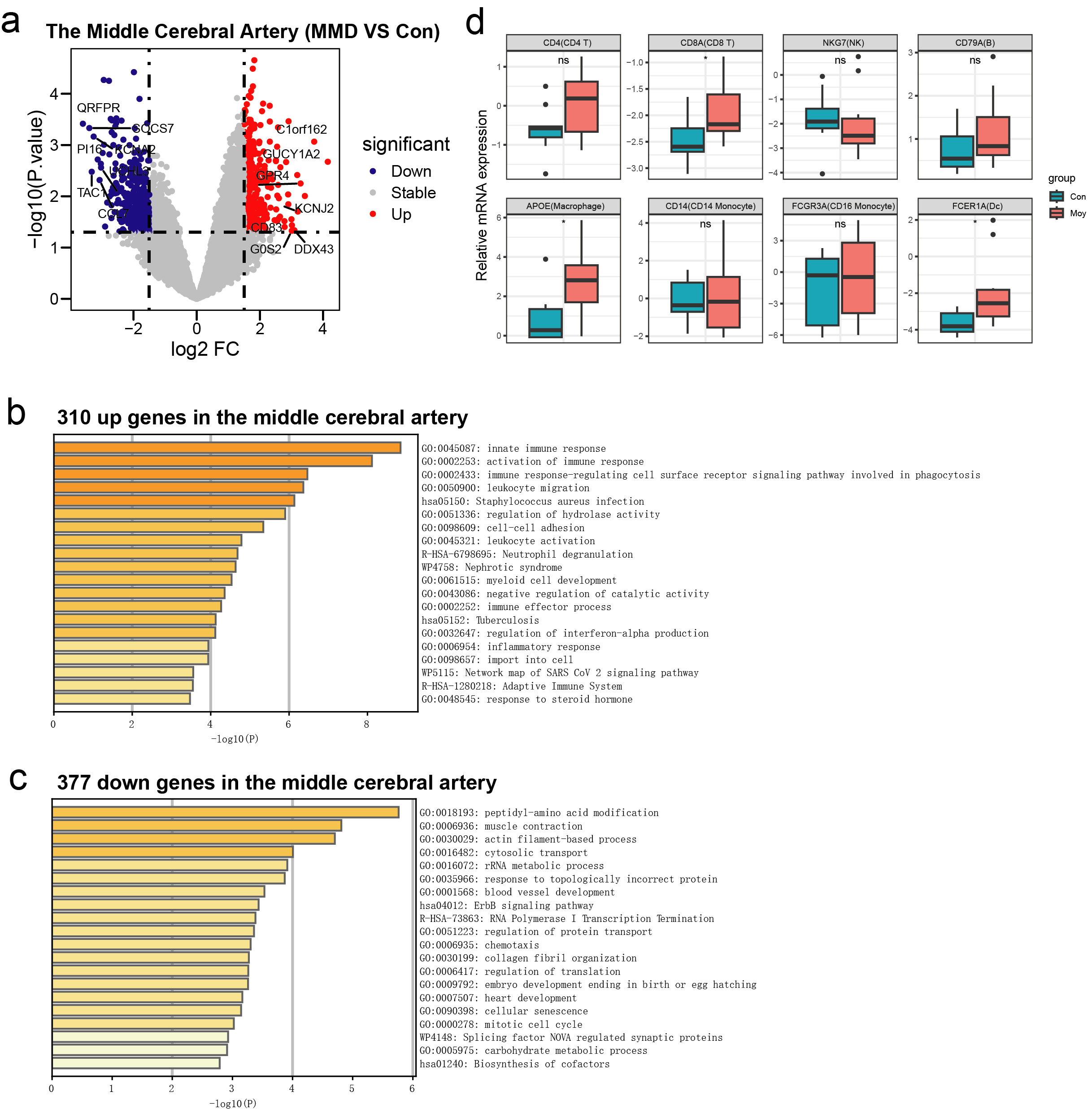

Supplement: Supplementary file 1 — Supplementary Material 1: Fig. 1: Abnormal Changes in the Middle Cerebral Arteries of Moyamoya Disease Patients. (a) A comparative differential expression analysis between the middle cerebral arteries of moyamoya disease patients and normal controls. (b) Functional enrichment analysis of the 310 upregulated genes in the middle cerebral arteries of moyamoya disease patients. (c) Functional enrichment analysis of the 377 downregulated genes in the middle cerebral arteries of moyamoya disease patients. (d) Differential expression of various immune cell markers between the middle cerebral arteries of moyamoya disease patients and controls. Con, controls. Moy, moyamoya disease. [file 13023_2026_4241_MOESM1_ESM.png]

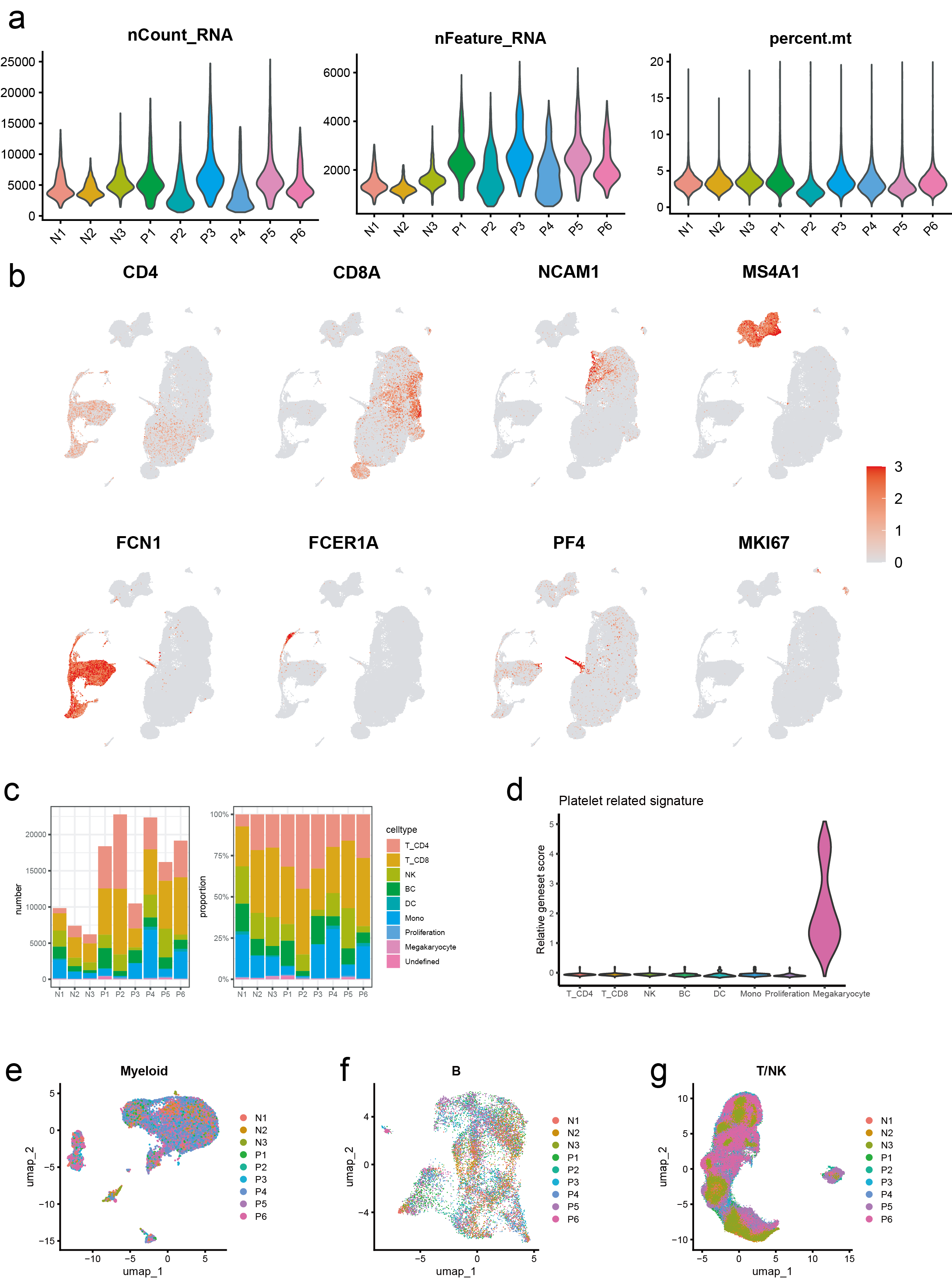

Supplement: Supplementary file 2 — Supplementary Material 2: Fig. 2: Peripheral Blood Single-Cell Data Preprocessing and Overview of Information. (a) Violin plots display the expression levels of UMI, Feature, and mitochondrial gene expression after quality control for each sample. (b) Feature plots illustrate the annotation markers for various cell types. (c) Absolute and relative proportions of various cell types across samples. (d) Violin plots of the platelet module score across cell types. (e-g) UMAP plots colored by sample origin. N, normal controls. P, patients. [file 13023_2026_4241_MOESM2_ESM.png]

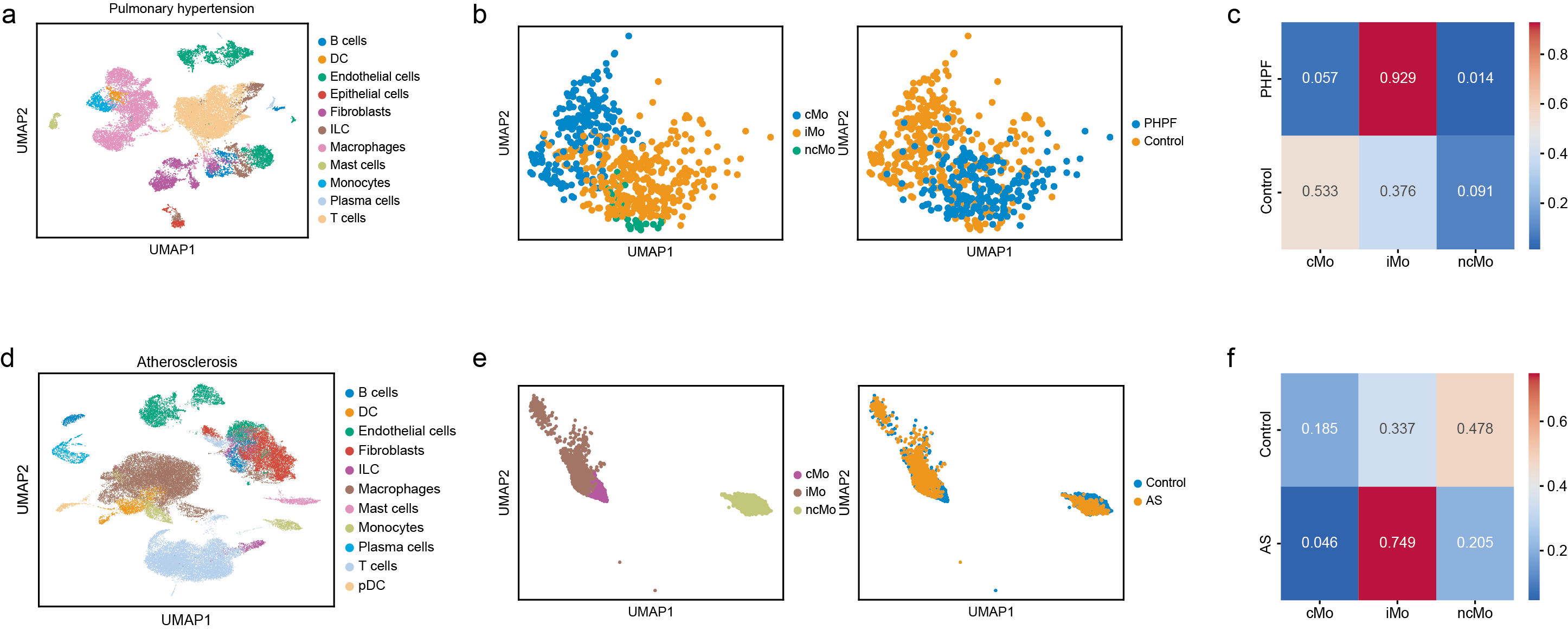

Supplement: Supplementary file 3 — Supplementary Material 3: Fig. 3: Analysis of Monocytes in Peripheral Blood in Pulmonary Artery Hypertension and Atherosclerosis. (a) and (d) Single-cell transcriptomic data from peripheral blood in patients with pulmonary artery hypertension and atherosclerosis. (b) and (e) Identification of heterogeneity in monocytes from peripheral blood in pulmonary artery hypertension and atherosclerosis. (c) and (f) Relative proportions of various monocyte subtypes in control and disease groups, including pulmonary artery hypertension or atherosclerosis. PHPF, pulmonary arterial hypertension. AS, atherosclerosis. cMo, classical monocytes. iMo, intermediate monocytes. ncMo, nonclassical monocytes. [file 13023_2026_4241_MOESM3_ESM.png]

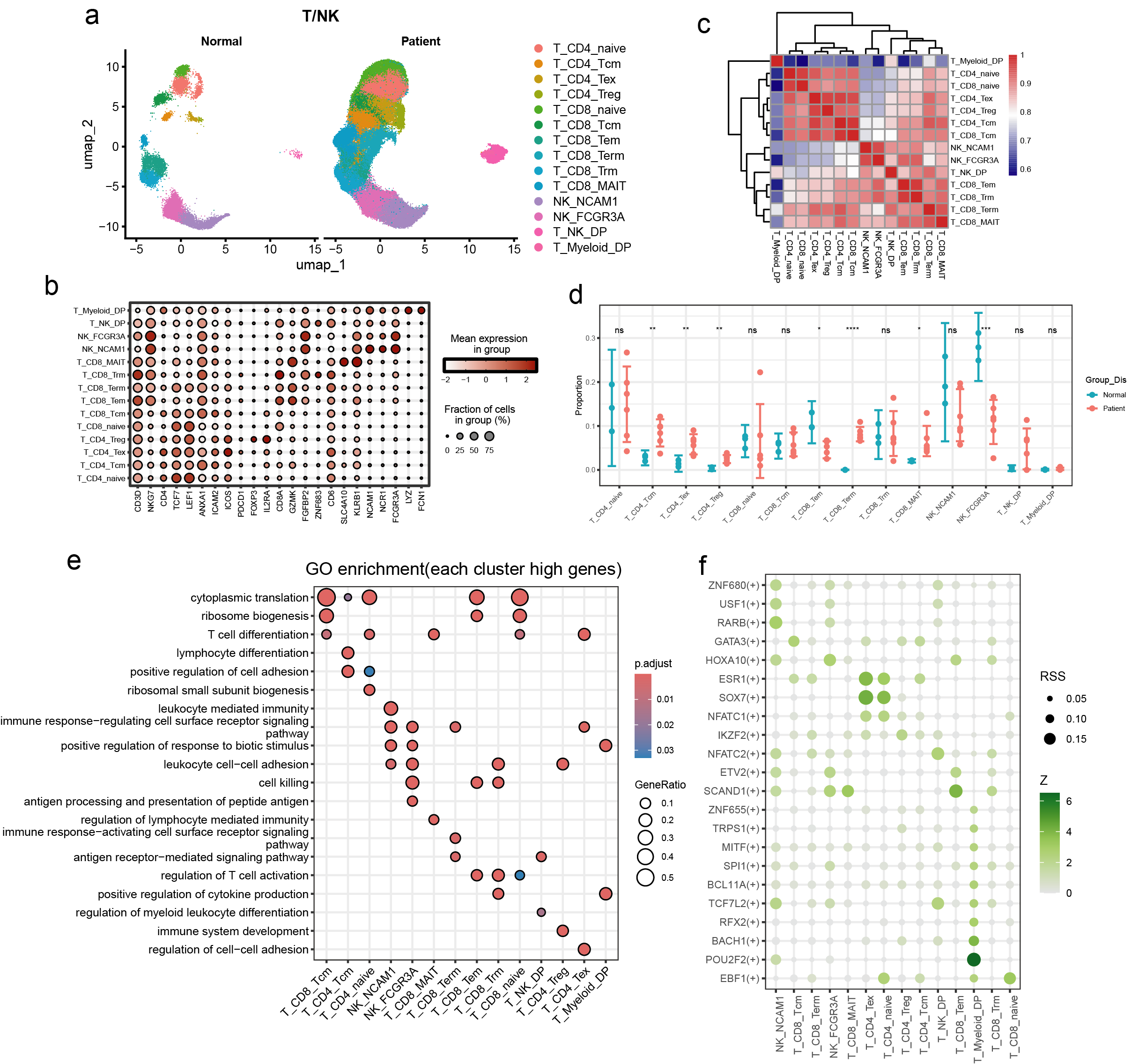

Supplement: Supplementary file 4 — Supplementary Material 4: Fig. 4: Single-Cell Analysis of Peripheral Blood T/NK Cells in Normal Controls and Moyamoya Disease Patients. (a) UMAP visualization of T/NK cell clustering in peripheral blood from normal and moyamoya disease groups. (b) Highly expressed genes across various T/NK cell subsets. (c) Heatmap of transcriptional pattern correlations among T/NK cell subsets. (d) Relative quantity differences of T/NK cell subsets between normal and moyamoya disease groups. Error bars represent the standard deviation. (e) GO enrichment analysis for highly expressed genes specific to each T/NK cell subset. (f) Specific activated transcription factors characteristic of various T/NK cell subsets. [file 13023_2026_4241_MOESM4_ESM.png]

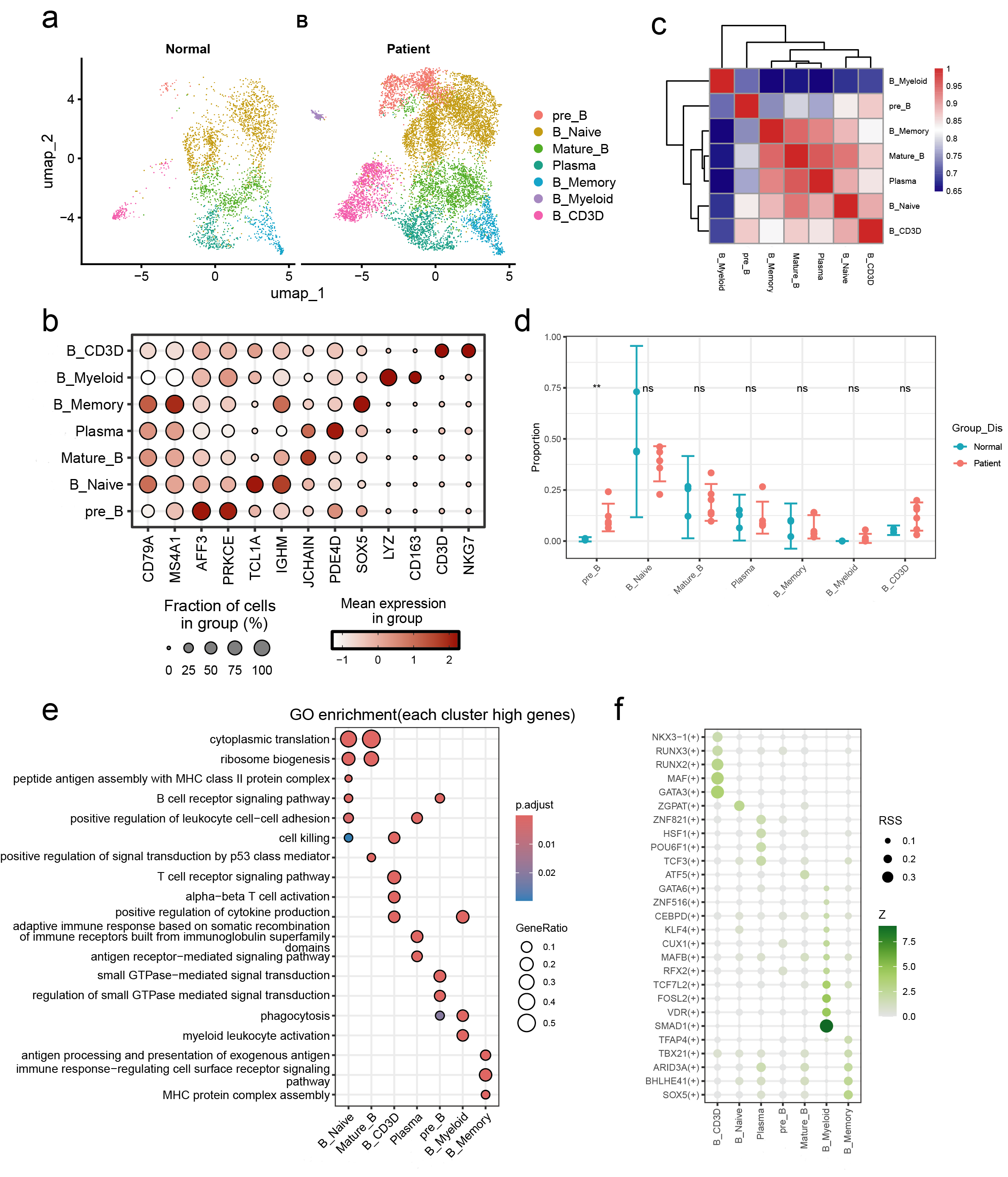

Supplement: Supplementary file 5 — Supplementary Material 5: Fig. 5: Single-Cell Analysis of Peripheral Blood B Cells in Normal and Moyamoya Disease Groups. (a) UMAP visualization of peripheral blood B cell clustering in normal and moyamoya disease groups. (b) Highly expressed genes in various B cell subsets. (c) Heatmap of transcriptional pattern correlations across B cell subsets. (d) Relative quantity differences of various B cell subsets between normal and moyamoya disease groups. Error bars represent the standard deviation. (e) GO enrichment analysis of highly expressed genes specific to each B cell subset. (f) Specific activated transcription factors in various B cell subsets. [file 13023_2026_4241_MOESM5_ESM.png]

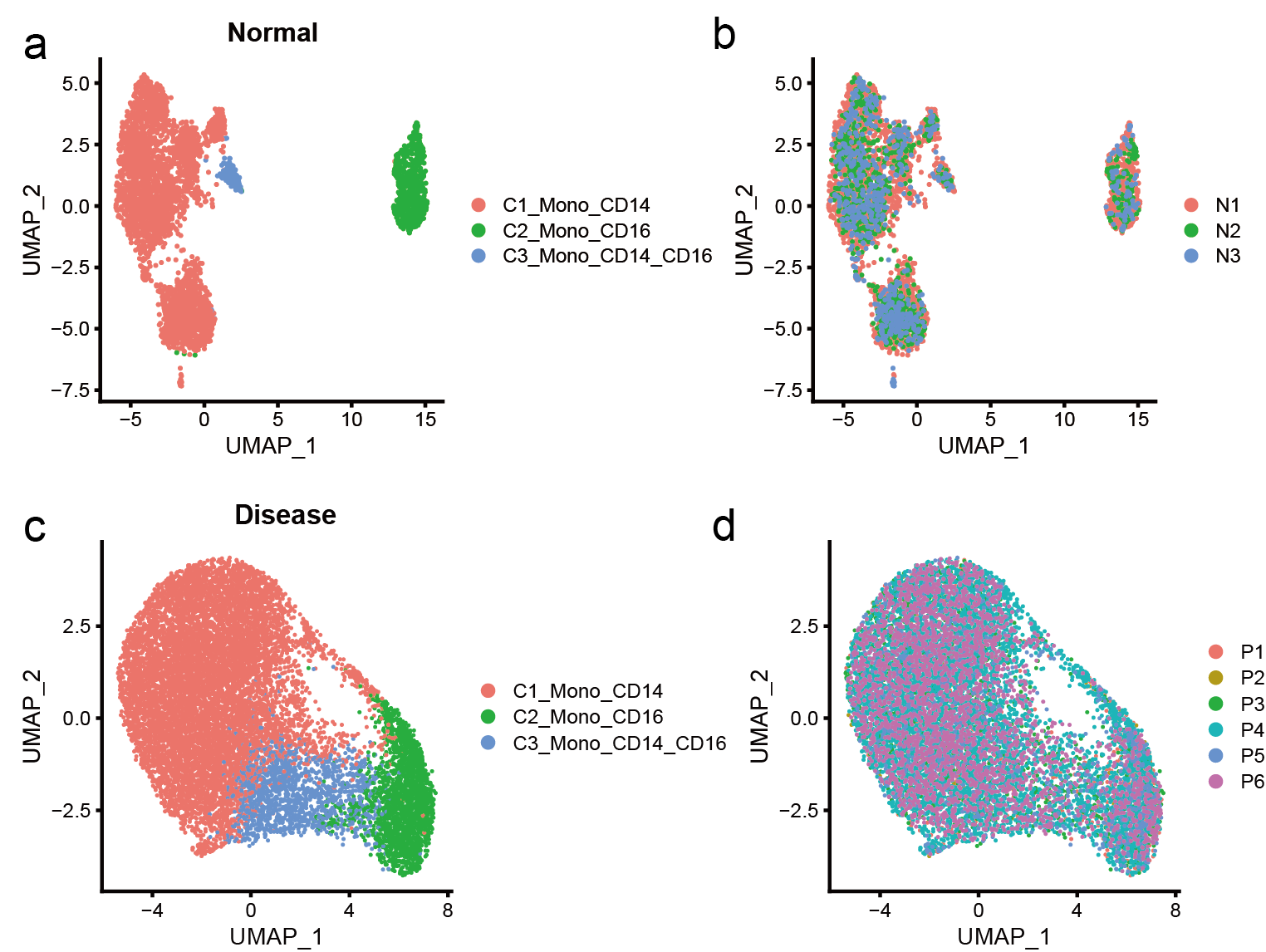

Supplement: Supplementary file 6 — Supplementary Material 6: Fig. 6: Trajectory analysis of monocyte differentiation in MMD and control groups. (a–d) UMAP projections showing the distribution and transitional continuum of monocyte subsets (CD14⁺, intermediate, and CD16⁺) in MMD patients (a, c) and healthy controls (b, d). Note the clear bridging population of intermediate monocytes in MMD, which is absent in controls. N, normal controls. P, patients. [file 13023_2026_4241_MOESM6_ESM.png]
